# Isolation and Identification of Indole Alkaloids from *Aspergillus amstelodami* BSX001 and Optimization of Ultrasound-Assisted Extraction of Neoechinulin A

**DOI:** 10.3390/microorganisms12050864

**Published:** 2024-04-26

**Authors:** Shuyao Li, Xiaobo Liu, Qiuya Gu, Xiaobin Yu

**Affiliations:** 1 The Key Laboratory of Industrial Biotechnology, Ministry of Education, School of Biotechnology, Jiangnan University, Wuxi 214122, China; lsy6210208025@163.com; 2 School of Environmental and Biological Engineering, Nanjing University of Science and Technology, 200 Xiaolingwei Street, Nanjing 210094, China; xbliu@njust.edu.cn

**Keywords:** *Aspergillus amstelodami*, indole alkaloids, antioxidant activity, neoechinulin A, extraction optimization

## Abstract

This study aimed to investigate the alkaloid secondary metabolites of *Aspergillus amstelodami* BSX001, a fungus isolated from Anhua dark tea, and to improve the extraction yield of the active ingredients by optimizing the extraction process. The structural characterization of the compounds was investigated using mass spectrometry (MS) and nuclear magnetic resonance (NMR) spectroscopy. The antioxidant activity of echinulin-related alkaloids was evaluated by determining the total reducing power and DPPH radical scavenging capacity. The extraction process of the compound with optimum activity was optimized by a single-factor test and response surface methodology (RSM) combined with Box–Behnken design (BBD). The optimized result was validated. Finally, a new alkaloid 8-hydroxyechinulin (**1**), and four known alkaloids, variecolorin G (**2**), echinulin (**3**), neoechinulin A (**4**), and eurocristatine (**5**), were isolated. Echinulin-related compounds **1**, **3**, and **4** possessed certain antioxidant activities, with IC_50_ values of 0.587 mg/mL, 1.628 mg/mL, and 0.219 mg/mL, respectively, against DPPH radicals. Their total reducing power at a concentration of 0.5 mg/mL was 0.29 mmol/L, 0.17 mmol/L, and 4.25 mmol/L. The extraction process of neoechinulin A was optimized with the optimum extraction parameters of 72.76% methanol volume fraction, 25 mL/g solid–liquid ratio, and 50.8 °C soaking temperature. Under these conditions, the extraction yield of neoechinulin A was up to 1.500 mg/g.

## 1. Introduction

The “Golden Flower” fungi are the dominant fungi in the flowering process of Fu-brick tea, which are composed of fungi of the genera *Aspergillus* sp. and *Eurotium* sp. Based on scientific research and updates to the classification system, *Eurotium* sp. is now classified in the genus *Aspergillus*. “Golden Flower” fungi can secrete a variety of active metabolites, which are key factors affecting the quality of tea, and have many functional benefits for human health, such as the prevention of hypertension and cardiovascular disease, having antioxidant and anti-inflammatory properties, and so on [1]. As their functional activities are continuously explored, the metabolites of “Golden Flower” fungi have also gained wider attention. “Golden Flower” fungi have gradually been applied to the fermentation of many raw materials, such as plant substitute teas, grains, medicinal herbs, etc., and have given the products new application values through fermentation [2]. At present, the reported secondary metabolites of “Golden Flower” fungi are mainly classified into indole diketopiperazine alkaloids, benzaldehyde derivatives, anthraquinone derivatives, and other compounds [3].

Indole diketopiperazine alkaloids are cyclic dipeptides containing indole structural units formed by the combination of two amino acids and are widely found in the secondary metabolites of *Penicillium* and *Aspergillus* fungi. The discovered indole diketopiperazine alkaloids currently include echinulins, tryprostatins, fumitremorgins, roquefortines, notoamieds, variecolortides, etc. [4]. The echinulin-related compounds are derived from cyclo-L-alanyl-L-tryptophan dipeptides, which undergo prenylation by two prenyltransferases, culminating in the formation of the trans-pentenyl group on the C-2 and the ortho-pentenyl group on the C-4-C-7 of the indole fraction [5]. Due to their significant antioxidant, antibacterial, and antiviral properties, echinulin-related compounds have become a research hotspot for chemists, pharmacists, and biologists [6]. Neoechinulin A is the most widely studied echinulin-related compound, with free radical scavenging activity and neuroprotective activity [7,8]. It can improve the memory function of lipopolysaccharide-treated mice and exert antidepressant-like effects by altering the 5-hydroxytryptamine system [9]. Neoechinulin A can treat inflammation in RAW264.7 macrophages by inhibiting the NF-κB and p38 MAPK pathways [10]. It can also prevent cervical cancer and so on [11]. Currently, echinulin-related compounds can be obtained through natural product separation and synthetic methods. However, the synthetic methods have high costs and low activity due to differences in the chemical stereo configurations of the products and the natural sources [12]. Most of the current studies focus on activity discovery, and there is a lack of quantitative assays for specific compounds, and few studies have focused on the extraction yield of echinulin-related compounds from natural sources. In this study, we used a strain of *Aspergillus amstelodami* BSX001 isolated from Anhua dark tea for solid state fermentation to separate and characterize indole diketopiperazine alkaloids in the fermentation products and obtained one new echinulin-related alkaloid and four known indole diketopiperazine alkaloids. The total reducing power and DPPH radical scavenging ability of echinulin-related compounds in the fermentation products were compared. A high-performance liquid chromatography (HPLC) assay was established for the most active neoechinulin A, and the extraction process was optimized by the design of a response surface test.

## 2. Materials and Methods

### 2.1. General Experimental Procedures

A Bruker DRX-500MHz NMR spectrometer (Bruker, Saarbrucken, Germany) was used to record NMR data. An LC–IT–TOF mass spectrometer (Shimadzu, Kyoto, Japan) was used to measure the mass spectral data. Semi-preparative HPLC was performed on a Waters 2535 semi-preparative liquid chromatograph (Waters, Milford, MA, USA) equipped with a diode array detector. Analytical HPLC was performed on an Agilent 1260 HPLC (Agilent, Santa Clara, CA, USA) equipped with a diode array detector. A Büchi rotary evaporator (Büchi, Flawil, Switzerland) was used for concentration operations. SHB-3 circulating multi-purpose vacuum pump (Yuhua Instrument Co., Ltd., Gongyi, China) was used. The UV spectra were determined using a ZF-1 triple-use UV meter (Shanghai Jingke Industrial Co., Ltd., Shanghai, China). Absorbance was measured using a microplate reader (BioTek, Winooski, VT, USA). Silica gel (80–100, 200–300 mesh, Qingdao Kangyexin Pharmaceutical Silica Gel Desiccant Co., Ltd., Qingdao, China), Sephadex LH-20 (Amersham Biosciences, Uppsala, Sweden), preparative reversed-phase chromatographic columns (250 mm × 20 mm, 10 μm, Daisogel, Osaka, Japan), and RP-C18 (40–63 μm, Merck, Darmstadt, Germany) were used for column chromatography. Thin-layer chromatography (TLC) was performed on GF254 silica gel plates (50–100 nm, Haixiang Chemical Co., Ltd., Linyi, China). All reagents used for HPLC were liquid phase grade and purchased from Sigma-Aldrich (Shanghai, China). 1,1-diphenyl-2-picrylhydrazide (DPPH) was purchased from Aladdin Biochemical Technology Co. (Shanghai, China). All other chemicals and reagents used were of analytical grade from commercial sources and purchased from Sinopharm (Shanghai, China). All experimental water was ultrapure water purified by a water purifier.

### 2.2. Fermentation of A. amstelodami BSX001

*A. amstelodami* BSX001 was isolated by the Laboratory of Fermentation and Healthy Foods, School of Bioengineering, Jiangnan University, and deposited in the Agricultural Culture Collection of China (ACCC) under the accession number AC-CC32729.

Potatoes were washed, peeled, and chopped. Then, 200 g of diced potatoes were weighed and added to 1000 mL of distilled water, boiled for about 30 min, and then filtered. The filtrate had 25 g of raw dark green tea added to it, was boiled for 5 min, and filtered again. Next, 20 g of glucose and 20 g of agar were added to the filtrate, and distilled water was fixed to 1000 mL to obtain the modified potato-dextrose agar (PDA) medium. *Eurotium amstelodami* BSX001 was inoculated in the modified PDA medium for 5 days at 30 °C and then transferred to Czapek’s medium (250 mL conical flask containing 100 mL of liquid) for 4 days at 30 °C at 200 r/min to prepare the seed solution. The seed solution was inoculated into the solid fermentation medium at 10% inoculum volume and incubated at 30 °C for 10 days.

Czapek’s medium: sucrose 3 g, NaNO_3_ 0.3 g, K_2_HPO_4_ 0.1 g, KCl 0.05 g, MgSO_4_·7H_2_O 0.05 g, FeSO_4_ 0.001 g, agar 1.8 g, distilled water 100 mL, natural pH.

Solid fermentation medium: rice 200 g, yeast extract 10 g, sea crystal 6 g, sucrose 8 g, bran 6 g, distilled water 60 mL.

### 2.3. Extraction and Isolation

After the fermentation, the medium was dried at 60 °C and then pulverized. Ethyl acetate has strong solubility, good stability, and environmental friendliness, making it suitable for the extraction and purification of most substances. The extraction of fermentation products was performed according to the method previously described with modifications [13]. The treated medium (3.1 kg) was mixed with ethyl acetate in a ratio of 1:2. Afterwards, the mixture was sonicated in an ultrasound cleaner for 120 min (40 kHz) at room temperature (25 °C). The ethyl acetate extract was filtered. The filtrate was collected and concentrated under reduced pressure to obtain the crude extract. The ultrasound extraction process was repeated three times and a total of 41.0 g of extract was combined. 

The size of the glass column for silica gel column chromatography is 400 mm × 50 mm, 400 mm × 20 mm, and the size of the glass column for gel column chromatography is 1500 mm × 10 mm. Dissolve the ethyl acetate extract, add 80–100 mesh silica gel, and mix the sample until the silica gel is gravelly, transfer the mixture to a rotary evaporation flask for distillation under reduced pressure and evaporate the solvent. Take an appropriate amount of 200–300 mesh silica gel, and add the solvent system stirring to fill the column, followed by the sample. The petroleum ether–acetone system was used as the mobile phase, and the gradient elution was carried out in 20:1, 15:1, 10:1, 5:1, 1:1 (*v*/*v*), and the fractions were monitored by thin-layer chromatography (TLC) using a GF254 silica gel plate, and then combined according to the results of UV chromatography as well as the heating chromatography results of vanillin sulfate to obtain a total of four fractions of Fr.1~Fr.4 finally. Fr.2 (2.1 g) was subjected to silica gel column chromatography with 100:1 (*v*/*v*) isocratic elution using a chloroform–methanol system as the mobile phase, then chromatographed on a Sephadex LH-20 gel column with isocratic elution using a mobile phase of chloroform:methanol = 1:1, and finally chromatographed on the semi-preparative HPLC (preparative reversed-phase column, 250 mm × 20 mm, 10 μm, Daisogel, Osaka, Japan) at a detection wavelength of 210 nm, isocratic elution was carried out with methanol:water = 75:25 as the mobile phase, and the sample with a retention time of 31.7 min was collected according to the liquid phase results, and compound **2** (12 mg) was obtained after rotary evaporation and drying. Fr.3 (3.0 g) was continued to be subjected to silica gel column chromatography, using a chloroform–methanol system as the mobile phase, 100:1 (*v*/*v*) isocratic elution. Then, the separation and purification were continued using the semi-preparative HPLC with methanol:water = 63:37 as mobile phase at a detection wavelength of 210 nm, and samples with a retention time of 14.8 min were collected based on the liquid phase results, recrystallized in methanol, after natural evaporation of the solvent, compound **4** (28 mg) was obtained. The column chromatography of Fr.4 (4.3 g) was continued on silica gel, using a chloroform–methanol system as the mobile phase, eluted according to a gradient of 60:1, 55:1, 50:1, 45:1, 40:1, 35:1, 30:1, 25:1, 20:1, 15:1, 10:1 (*v*/*v*), and the fractions Fr.4-1~4-3 were obtained by combining components after detection by TLC. Compound **5** (19 mg) was obtained from Fr.4-1 using Sephadex LH-20 gel column chromatography with chloroform:methanol=1:1 as mobile phase, isocratic elution, and dried by rotary evaporation. Fr.4-2 was evaporated to dryness and washed with methanol to evaporate naturally to obtain compound **1** (10 mg). Fr.4-3 was evaporated to dryness and washed with methanol to evaporate naturally to obtain compound **3** (180 mg).

### 2.4. DPPH Radical Scavenging Assay

DPPH assays were performed according to the method previously described with minor modifications [14]. In a 96-well microtiter plate, samples (50 μL) dissolved in DMSO at concentrations of 4, 2, 1, 0.5, 0.25, 0.125, 0.0625, and 0.03125 mg/mL were added to a DPPH solution (150 μL) dissolved in EtOH at a concentration of 0.02 mM and mixed well. The mixture was incubated at 37 °C for 60 min. The absorbance was measured at 510 nm with a microplate reader and the inhibition rate was calculated according to Equation (1). Vitamin C was used as a positive control, and DMSO was used as a negative control.

(1) $$Y=\left(1-\frac{A-B}{C-D}\right)\times 100\%$$

where Y is the DPPH radical scavenging rate (%), and A, B, C, and D denote the absorbance of the sample group, sample blank group, the control group, and the control blank group, respectively.

### 2.5. Total Reducing Power Assay

The total reducing power assay was performed according to the method previously described with minor modifications [15]. The sample solution (0.125 mL), 1% potassium ferricyanide solution (0.125 mL), and 0.1 mol/L PBS (0.125 mL) were mixed well and reacted in a water bath at 50 °C for 20 min and then rapidly cooled. Next, 10% trichloroacetic acid solution (0.125 mL) was added to the reaction system and mixed well, and the mixture was centrifuged (10,000 rpm, 5 min). The supernatant (0.25 mL), water (0.25 mL), and 0.1% ferric chloride solution (0.25 mL) were mixed well, and the absorbance value was measured at 700 nm with a microplate reader after 10 min at room temperature (25 °C). The standard curve was prepared according to the above method with 0.125, 0.250, 0.375, 0.500, and 0.625 mmol/L L-cysteine hydrochloride as the standard solution. It was y = 0.6142x + 0.1563 and R^2^ = 0.9987, where x was the content of L-cysteine hydrochloride (mmol/L) and y was the absorbance value at 700 nm.

### 2.6. Determination of Neoechinulin A in Fermentation Products by HPLC Method

The HPLC method was used for the determination of neoechinulin A. Since neoechinulin A is soluble in methanol, dissolving the sample in methanol has better compatibility with the mobile phase, which is more conducive to the separation of the substances, and good liquid-phase detection results can be obtained. Therefore, methanol was used as the solvent when optimizing the extraction conditions of neoechinulin A. The dried and pulverized fermentation medium was mixed proportionally with methanol (HPLC grade). The mixture was soaked for 30 min at a specific temperature and then placed in an ultrasound cleaner for sonication (40 kHz). The mixture was centrifuged (8000 rpm, 5 min) at the end of extraction. The supernatant was filtered through the 0.22 μm ultrafiltration membrane and subjected to HPLC.

The neoechinulin A standard and fermentation samples were quantified using an Agilent 1260 HPLC system (Agilent Technologies, Santa Clara, CA, USA) equipped with a ZORBAX Eclipse Plus C18 column (250 mm × 4.6 mm, 5 μm). Standard solutions of neoechinulin A were prepared in methanol (HPLC grade) at concentrations of 0.02, 0.04, 0.06, 0.08, and 0.10 mg/mL. Isocratic elution was performed using acetonitrile (solvent A) and 0.1% phosphoric acid aqueous solution (solvent B) as mobile phases (A:B = 3:7, *v*/*v*). The mobile phase flow rate was 1 mL/min, the column temperature was 30 °C, and the injection volume was 10 μL, and it was monitored by a UV spectrophotometric detector with an absorbance of 254 nm. The standard curve of neoechinulin A content was determined using the following equation: y = 11414x − 3.9905, R^2^ = 0.9986.

### 2.7. Single-Factor Test

The single-factor tests were carried out to determine the optimum range of extraction conditions for neoechinulin A by varying one of the variables and fixing the other factors. The extraction yield of neoechinulin A was the response variable of the single-factor experiments, and was calculated according to Equation (2).

(2) $$Y=\frac{C\times V\times N}{W}$$

Y is the extraction yield of neoechinulin A, mg/g; C is the concentration of neoechinulin A obtained by the HPLC method, mg/mL; V is the total volume of extract, mL; N is the dilution factor; and W is the weight of the material, g.

Single-factor optimization tests were conducted sequentially for methanol volume fraction, solid–liquid ratio, soaking temperature, and ultrasound time. The initial conditions of extraction were 100% methanol volume fraction, solid–liquid ratio of 20 mL/g, soaking temperature of 20 °C, and ultrasound time of 120 min.

With the constant control of solid–liquid ratio, soaking temperature, and ultrasound time, the fermentation products were extracted by methanol with the volume fractions of 30%, 40%, 50%, 60%, 70%, 80%, 90%, and 100%, and the optimal methanol fraction was determined.

The methanol volume fraction, soaking temperature, and ultrasound time were kept constant, and the fermentation products were extracted using the solid–liquid ratios of 5 mL/g, 10 mL/g, 15 mL/g, 20 mL/g, 25 mL/g, 30 mL/g, 35 mL/g, and 40 mL/g. The optimal solid–liquid ratio was determined.

The methanol volume fraction, solid–liquid ratio, and ultrasound time were controlled to be unchanged, and the fermentation products were extracted at 20 °C, 30 °C, 40 °C, 50 °C, 60 °C, 70 °C, 80 °C, and 90 °C, and the optimal soaking temperature was determined.

The methanol volume fraction, solid–liquid ratio, and soaking temperature were controlled to be unchanged, and the ultrasound time was set to 20 min, 40 min, 60 min, 80 min, 100 min, and 120 min, and the optimal ultrasound time was determined.

### 2.8. Box–Behnken Design

A three-factor, three-level BBD model was used to select the optimal extraction conditions. The three factors were methanol volume fraction, solid–liquid ratio, and soaking temperature. Seventeen experiments were designed using Design Expert 8.0.6 software and the extraction experiments were conducted in randomized batches. Analysis of variance (ANOVA) was used to determine the applicability of the proposed model. The coefficient of determination (R^2^, adjusted R^2^, and predicted R^2^) was used to evaluate the quality of fit of the model.

### 2.9. Statistical Analysis

All experiments were repeated three times and data were analyzed using SPSS 25.0. Values were shown as mean ± standard deviation of three replications. One-way ANOVA was used to analyze the significance of differences. Different letters indicated significant differences between each other (*p* < 0.05). Graphs were generated using Origin 9.5.

## 3. Results and Discussion

### 3.1. Structural Identification of Compounds

**Figure 1:**
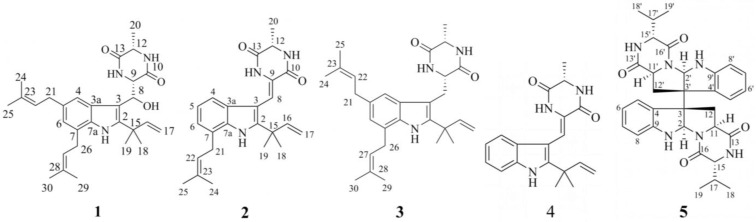
Chemical structures of compounds **1**–**5**.

The ethyl acetate soluble fraction of the solid fermentation product of *A. amstelodami* BSX001 was purified by silica column chromatography, Sephadex LH-20 chromatography, and preparative high-performance liquid chromatography. One new echinulin-related alkaloid (**1**) was obtained, and four known alkaloids, variecolorin G (**2**), echinulin (**3**), neoechinulin A (**4**), and eurocristatine (**5**), were obtained (Figure 1). The structures were analyzed as follows.

Compound **1**: White powder, ESI–MS *m*/*z*: 500 [M + Na]^+^. Table 1 showed the NMR characterization of compound **1**. ^13^C NMR and DEPT patterns showed seven methyl groups, three methylene groups, eight methine groups, and eleven quaternary carbons (including two ester carbonyl carbons, eight aromatic or olefinic carbons, and one alkane carbon). ^1^H NMR (500 MHz, CDCl_3_) showed signals, *δ*_H_: 1.88 (3H, s), 1.82 (3H, s), 1.73 (6H, m), 1.52 (3H, s), 1.50 (3H, s), and a bimodal methyl proton, *δ*_H_: 1.51 (3H, d, *J* = 6.9 Hz). Combining the ^1^H and ^13^C NMR patterns it can be hypothesized that the structure contains two isopentenyl groups [*δ*_H_: 5.34/5.43 (each 1H, m), 3.37/3.53 (each 2H, d, *J* = 7.3 Hz), 1.88/1.83 (each 3H, s), 1.73/1.72 (6H); *δ*_C_: 34.7/31.5, 124.6/123.0, 131.4/132.9, 17.9/17.9, 25.7/25.8], a monosubstituted double bond [*δ*_H_: 6.12 (1H, dd, *J* = 17.4, 10.6 Hz), 5.21 (1H, dd, *J* = 10.6, 0.7 Hz), 5.15 (1H, dd, *J* = 17.4, 0.7 Hz); *δ*_C_: 145.6, 112.6], two ester carbonyls [*δ*_C_: 167.0, 170.1]. Based on the above signal characteristics and considering the source of the compound, it can be hypothesized that it is an indole diketopiperazine alkaloid. Further analysis of the NMR data reveals that it is very similar to echinulin, an alkaloid originating from *Eurotium rubrum* [16]. Methylene signal in echinulin is changed to downfield shifted methine signal in **1**, thus, it is hypothesized that compound **1** is a derivative of echinulin with 8-position hydroxyl substitution.

**Figure 2:**
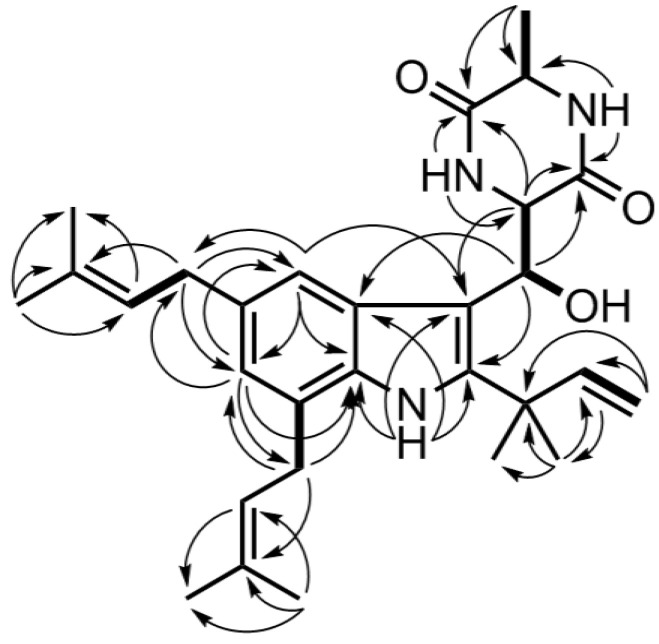
Key COSY (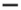) and HMBC (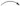) correlations for Compound **1**.

The correlation of H-8 (*δ*_H_ 5.46) with C-2 (*δ*_C_ 143.1), C-3 (*δ*_C_ 106.1), C-3a (*δ*_C_ 126.8), C-9 (*δ*_C_ 56.0), and C-10 (*δ*_C_ 170.1) and the correlation of 8-OH (*δ*_H_ 4.95) with C-3, C-8 (*δ*_C_ 69.4), and C-9 in HMBC verified the above speculation. Further analysis of 2D NMR (Figure 2) provided further validation of the overall structure. Firstly, the HMBC correlation of H-4 (*δ*_H_ 7.41) with C-3a, C-3, C-6 (*δ*_C_ 123.1), C-7a (*δ*_C_ 132.6), and C-21 (*δ*_C_ 34.7), the HMBC correlation of H-6 (*δ*_H_ 6.79) with C-4 (*δ*_C_ 117.2), C-7a, C-21, and C 26 (*δ*_C_ 31.5), the HMBC correlation of H-21 (*δ*_H_ 3.37) with C-4 (*δ*_C_ 117.2) and C-6, the correlation of H-26 (*δ*_H_ 3.37) with C-6 and C-7a, and the HMBC correlation of 1-*N*H (*δ*_H_ 8.15) with C-2, C-3, C-3a, and C-7a verified the structural fragment of the indole parent nucleus and the substitution position of the isopentenyl group on the benzene ring. Secondly, the HMBC correlation of H-18 (*δ*_H_ 1.52) with C-2, C-15 (*δ*_C_ 38.7), C-16 (*δ*_C_ 145.6), and C-19 (*δ*_C_ 28.3), the HMBC correlation of H-17 (*δ*_H_ 5.21, 5.15) with C-15 and C-16, and the ^1^H-^1^H COSY correlation of H-16 (*δ*_H_ 6.12) with H-17 verified the structural fragment with the two-position substituent. Thirdly, the HMBC correlation of H-9 (*δ*_H_ 4.66) with C-10 and C-13, the HMBC correlation of 11-*N*H (*δ*_H_ 5.34) with C-9, C-12, and C-13, the HMBC correlation of 14-*N*H (*δ*_H_ 6.14) with C-9 and C-13, the HMBC correlation of H-20 (*δ*_H_ 1.51) with C-12 (*δ*_C_ 50.6) and C-13 (*δ*_C_ 167.0), and the ^1^H-^1^H COSY correlation of H-20 with H-12 (*δ*_H_ 4.13) validated the structural unit of diketopiperazine. In addition, the HMBC correlation of H-9 with C-3, the HMBC correlation of H-8 with C-2, C-3, C-3a, and C-10, and the COSY correlation of H-8 with H-9 validated the connection method from C-3 to C-8 and then to C-9. Finally, the HMBC correlation of H-24 (*δ*_H_ 1.73) with C-22 (*δ*_C_ 124.6), C-23 (*δ*_C_ 131.4), and C-25 (*δ*_C_ 25.8), the ^1^H-^1^H COSY correlation of H-21 with H-22, the HMBC correlation of H-29 (*δ*_H_ 1.88) with C-27 (*δ*_C_ 123.0), C-28 (*δ*_C_ 132.0), and C-30 (*δ*_C_ 25.7), and the ^1^H-^1^H COSY correlation of H-26 with H-27 verified the structural units of the two isopentenyl groups. In summary, the structure of compound **1** was established (Figure 1(1)) and named 8-hydroxyechinulin.

Compound **2**: White powder, ESI–MS *m*/*z*: 414 [M + Na]^+^. Based on the NMR characterization (Table S1), combined with previous reports [17], compound **2** was identified as variecolorin G.

Compound **3**: White powder, ESI–MS *m*/*z*: 461 [M^+^]. Based on the NMR characterization (Table S2), combined with previous reports [16], compound **3** was identified as echinulin.

Compound **4**: Yellow powder, ESI–MS *m*/*z*: 346 [M + Na]^+^. Based on the NMR characterization (Table S3), combined with previous reports [18], compound **4** was identified as neoechinulin A.

Compound **5**: White powder, ESI–MS *m*/*z*: 591 [M + Na]^+^. Based on the NMR characterization (Table S4), combined with previous reports [19], compound **5** was identified as eurocristatine.

### 3.2. Determination of Antioxidant Activity

The occurrence of oxidative reactions is closely related to many diseases, such as aging, cancer, and cardiovascular diseases. Some studies have shown that echinulin-related compounds have good antioxidant activity [20]. The DPPH radical scavenging capacity and total reducing power of three echinulin-related alkaloids isolated from fermentation products were determined. As shown in Figure S20a, the DPPH radical scavenging rate of compound **1**, echinulin (**3**), and neoechinulin A (**4**) increased continuously with increasing concentration, and their DPPH radical scavenging rate was 87.88%, 81.82%, and 97.16% at a concentration of 2 mg/mL, respectively. As shown in Table 2, compound **1**, echinulin (**3**), and neoechinulin A (**4**) showed certain scavenging activity against DPPH radicals, and their IC_50_ values were 0.587 mg/mL, 1.628 mg/mL, and 0.219 mg/mL, respectively. The total reducing power of compound **1**, echinulin (**3**), and neoechinulin A (**4**) was 0.29 mmol/L, 0.17 mmol/L, and 4.25 mmol/L at a concentration of 0.5 mg/mL, respectively. Among the three echinulin-related alkaloids, neoechinulin A (**4**) showed the best antioxidant activity, which is consistent with previous reports [21,22]. The presence of the C-8/C-9 double bond contributed to its antioxidant activity [23]. In addition, it has been shown that the C-8/C-9 double bond of neoechinulin A (**4**) is also associated with many activities such as cytoprotective effects [24]. The DPPH radical scavenging and total reducing power of the new compound 8-hydroxyechinulin (**1**) was better than that of echinulin (**3**), and considering the similarity between its chemical structure and that of echinulin (**3**) (Figure 1), it was hypothesized that the OH group on C-8 played a role.

### 3.3. Optimization for Ultrasound-Assisted Extraction of Neoechinulin A 

**Figure 3:**
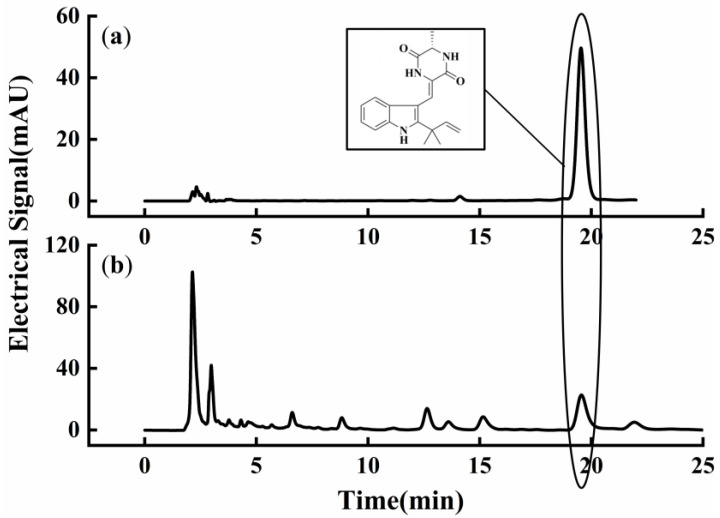
HPLC chromatograms. (**a**) Neoechinulin A standard, (**b**) *A. amstelodami* BSX001 fermentation sample.

Compound **4**, neoechinulin A, is the most widely studied echinulin-related compound. In addition to its antioxidant activity, it also possesses anti-inflammatory, antidepressant, and other biological activities, and has good application value [21]. The fermentation product of *A. amstelodami* BSX001 was extracted with methanol, and neoechinulin A in the fermentation samples was quantified by the HPLC method as shown in Figure 3. The parameters such as methanol volume fraction, solid–liquid ratio, soaking temperature, and ultrasound time, which affect the extraction yield of neoechinulin A, were optimized using a single-factor test and RSM with BBD. The optimization results were as follows:

#### 3.3.1. Optimization Results with Single-Factor Test 

**Figure 4:**
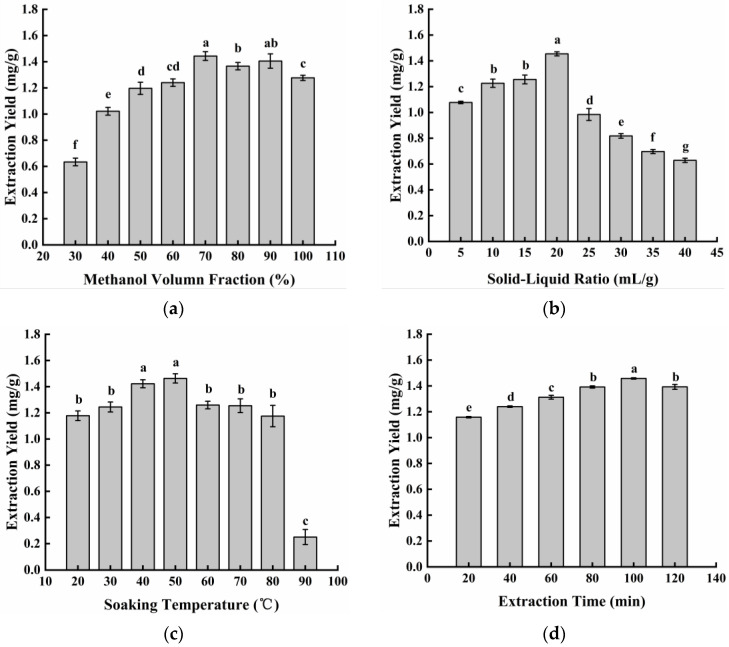
Effect of different factors on the extraction yield of neoechinulin A. (**a**) Methanol volume fraction, (**b**) material–liquid ratio, (**c**) soaking temperature, and (**d**) ultrasound time. Values are expressed as mean ± SD of three replicates. One-way ANOVA was used to assess the statistical significance of differences in expression levels. Different letters (a–g) indicate statistically significant differences (*p* < 0.05).

As shown in Figure 4a, the methanol volume fraction had a significant impact on the extraction efficiency. Although neoechinulin A is insoluble in water, appropriate moisture can swell the matrix and increase the contact surface area between the matrix and solvent, thus improving the extraction yield [25]. When the methanol volume fraction was increased to 70%, the extraction yield of neoechinulin A was highest, which was 1.443 mg/g. The extraction yield tended to decrease slowly at methanol volume fractions greater than 70%. This is probably because the vapor pressure, viscosity, and surface tension of 70% methanol solution are more favorable for the extraction of neoechinulin A from fermentation products. As the methanol volume fraction increases, the polarity of the solution changes and more impurities are dissolved, which may lead to a decrease in the extraction yield of neoechinulin A [26].

From Figure 4b, it can be seen that with the increase in the solid–liquid ratio, the extraction yield of neoechinulin A first increased and then decreased. The highest extraction yield reached 1.454 mg/g at the solid–liquid ratio of 20 mL/g. An appropriate increase in the solid–liquid ratio promoted the dissolution of the substance more efficiently. When the solid–liquid ratio reached a certain value, the neoechinulin A had already been completely dissolved, and a further increase in the solid–liquid ratio led to more impurities being dissolved, which affected the extraction yield of neoechinulin A [27].

From Figure 4c, it can be seen that the extraction yield of neoechinulin A increased with the increase in temperature and reached the maximum value of 1.463 mg/g at 50 °C. The increase in temperature led to the acceleration of the molecular movement and the enhancement of the diffusivity, which was favorable for the solubilization of neoechinulin A [28]. However, it also led to an increase in the dissolution of other impurities and finally resulted in a decrease in the extraction yield of neoechinulin A. In addition, as the soaking temperature increased, neoechinulin A was lost with the inevitable evaporation of methanol from the solvent.

As can be seen in Figure 4d, the extraction yield of neoechinulin A increased with increasing ultrasound time, reaching a maximum value of 1.458 mg/g at 100 min. When the ultrasound time was longer than 100 min, the extraction yield of neoechinulin A decreased. This may be attributed to the large solid–liquid concentration difference and high diffusion driving force at the beginning of the extraction process. However, when the concentration of neoechinulin A in the extraction solution and the material reached a dynamic equilibrium, the extraction yield remained constant [29]. In addition, if the ultrasound time was too long, the amount of impurities dissolved would also be increased to a certain extent, making the extraction yield of neoechinulin A decrease.

#### 3.3.2. Response Model Establishment

Based on the results of the single-factor test, methanol extraction fraction (A), material–liquid ratio (B), and soaking temperature (C) were used as the influencing factors in the response surface test according to the design principle of the Box–Behnken central combination test (Table 3).

The neoechinulin A extraction yield (mg/g) was used as the response value for the designed experiment. A three-factor, three-level BBD model was used and the results are shown in Table 4.

#### 3.3.3. Response Surface Analysis

The results of the above experiments were analyzed through Design Expert 8.0.6 software and the results are presented in Table 5.

The model was statistically significant (*p* < 0.0001). The *p*-value of the lack-of-fit item was 0.7788 (*p* > 0.05), which had a non-significant effect, indicating that the regression model could predict the experimental results well. The regression coefficient of the model, R^2^, was 0.9601, indicating that the model explained 96.01% of the variation in the total response value, and the value of R^2^_Adj_ was 0.9088. The values of R^2^ and R^2^_Adj_ were both close to 1, which suggested that the data had a high fit to the regression model [30]. The coefficient of variation (C.V.) was 2.38%, which was below the threshold of 10%, indicating that the model had strong experimental stability [31]. Therefore, the model can effectively analyze and predict the experimental results. In addition, the linear terms A and B, the quadratic terms A^2^ and C^2^, and the interaction terms AB and AC had *p*-values less than 0.05 and had a significant effect on neoechinulin A. The effects of the other factors were not significant.

After regression fitting, a quadratic regression model was obtained for the response values of the factors as follows:

(3) $$\begin{array}{cc} Y=-8.1007 & +0.20376A-0.027492B+0.095349C+0.000839521AB\\ & \;\;-0.000540036AC+0.000154872BC-0.00133112A^{2}\\ & \;\;-0.000769935B^{2}-0.000616854C^{2} \end{array}$$

where Y is the yield of neoechinulin A and A, B, and C are the coded values for the methanol volume fraction, solid–liquid ratio, and soaking temperature, respectively.

**Figure 5:**
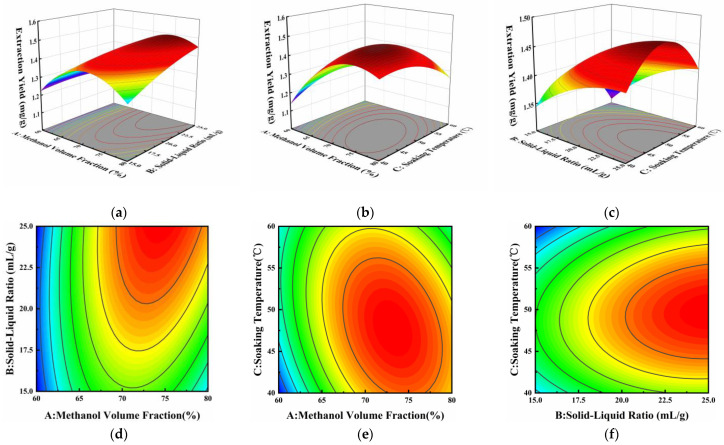
Effect of methanol volume fraction (A), solid–liquid ratio (B), and soaking temperature (C) on the extraction yield of neoechinulin A. Response surface plots (**a**–**c**) and contour plots (**d**–**f**) showed the effects of methanol volume fraction, solid–liquid ratio, and soaking temperature on the extraction yield of neoechinulin A.

Figure 5 illustrates the interaction between any two variables while holding the other variable at a fixed intermediate level. The interaction between variables is more pronounced as the curve of the three-dimensional (3D) response surface becomes steeper and the curve of the two-dimensional (2D) contour becomes closer to an ellipse. Combined with the analysis of variance (ANOVA) in Table 5, it can be seen that the interaction terms AB and AC had significant effects on the extraction yield of neoechinulin A, and the interaction term BC was not significant. As shown in Figure 5a,d, at a certain solid–liquid ratio, the extraction yield of neoechinulin A showed a tendency to increase and then decrease with the increase in the methanol volume fraction. Especially at high solid–liquid ratios, the increasing trend of extraction yield became more pronounced with the increase in methanol volume fraction. The large inclination angle and steep slope of the response surface indicate a significant interaction between methanol volume fraction and solid–liquid ratio [31]. Similarly, the interaction of methanol volume fraction and soaking temperature had a significant effect on the extraction yield of neoechinulin A as can be seen in Figure 5b,e.

#### 3.3.4. Optimal Conditions and Model Validation

Based on the above results, the optimal extraction conditions for neoechinulin A in the fermentation product of *A. amstelodami* BSX001 were obtained: methanol extraction fraction was 72.76%, solid–liquid ratio was 25 mL/g, and soaking temperature was 50.75 °C. The model predicted that the maximum extraction yield of neoechinulin A was 1.490 mg/g. Considering the limitations of experimental conditions, the extraction conditions were changed to 72.76% in methanol extraction fraction, 25 mL/g in solid–liquid ratio, and 50.8 °C in soaking temperature. To verify the feasibility of the model, three sets of validation tests were conducted under these conditions, and the extraction yield of neoechinulin A was 1.500 ± 0.026 mg/g. The relative error between the predicted and measured values was 0.69%, which was in high agreement. This indicates that the response surface model is suitable for the optimization of neoechinulin A extraction conditions.

## 4. Conclusions

In this study, the secondary metabolites of *A. amstelodami* BSX001 were analyzed, and a new indole diketopiperazine alkaloid, 8-hydroxyechinulin (**1**), and four known alkaloids were identified. In subsequent antioxidant experiments, 8-hydroxyechinulin exhibited better DPPH radical scavenging and total reducing power than echinulin (**3**), which is structurally similar to it. This was related to the OH group on C-8. In this study, neoechinulin A (**4**) was extracted by ultrasound-assisted methanol extraction. The optimal extraction conditions were determined by single-factor and BBD experiments: methanol extraction fraction of 72.76%, solid–liquid ratio of 25 mL/g, and soaking temperature of 50.8 °C. Under these conditions, the extraction yield of neoechinulin A could reach 1.500 mg/g. In summary, this study further expanded the structural diversity of secondary metabolites produced by *A. amstelodami* BSX001. A quantitative detection method for neoechinulin A was established, and a reasonable response surface model was established to optimize the extraction process of neoechinulin A in metabolites. This provided a certain reference significance for the mining of active compounds in *A. amstelodami* as well as the quantitative detection of echinulin-related compounds. In addition, neoechinulin A, the main subject of this study, in addition to its antioxidant activity, also possesses neurocytoprotective, anti-inflammatory, antitumor, antidepressant, and antimicrobial activities. It has been shown that neoechinulin A inhibits SIN-1-induced activation of caspase-3-like proteases and increases NADH-dehydrogenase activity, which helps to prevent neuronal cell death in neurodegenerative diseases [32]. Neoechinulin A can induce apoptosis in human cervical cancer Hela cells through down-regulation of Bcl-2 expression, up-regulation of Bax expression, and activation of the caspase-3 pathway [11]. In addition, it may be a potential therapeutic agent for the treatment of various inflammatory diseases [33]. Currently, there are relatively few studies on the relationship between the different activities of neoechinulin A. The next step could be to consider conformational relationship studies and investigate the linkage between the different activities of neoechinulin A through cellular as well as clinical trials to prepare for its further development and application.

## Figures and Tables

**Table 1:** NMR spectroscopic data of compound **1** in CDCl_3_ (*δ* in ppm).

No.	*δ*_C_ ^a^	*δ*_H_ ^b^ (J in Hz)	HMBC (H → C)
1-NH	-	8.15, s	C-2, C-3, C-3a, C-7a
2	143.1, qC	-	-
3	106.1, qC	-	-
3a	126.8, qC	-	-
4	117.2, CH	7.41, br. s	C-3, C-3a, C-6, C-7a, C-21
5	134.1, qC	-	-
6	123.1, CH	6.79, br. s	C-4, C-7a, C-21, C-26
7	123.5, qC	-	-
7a	132.6, qC	-	-
8	69.4, CH	5.46, dd (9.2, 1.0)	C-2, C-3, C-3a, C-9, C-10
8-OH	-	4.95, d (1.0)	C-3, C-8, C-9
9	56.0, CH	4.66, br. d (9.2)	C-3, C-8, C-9, C-13
10	170.1, qC	-	-
11-NH	-	5.34, overlap	C-9, C-10, C-12, C-13
12	50.6, CH	4.13, br. q (6.9)	C-13, C-20
13	167.0, qC	-	-
14-NH	-	6.14, overlap	C-9, C-13
15	38.7, qC	-	-
16	145.6, CH	6.12, dd (17.4, 10.6)	C-2, C-15, C-18, C-19
17	112.6, CH_2_	5.21, dd (17.4, 0.7)	C-15, C-16
		5.15, dd (10.6, 0.7)	
18	28.1, CH_3_	1.52, s	C-2, C-15, C-16, C-19
19	28.3, CH_3_	1.50, s	C-2, C-15, C-16, C-18
20	19.3, CH_3_	1.51, d (6.9)	C-12, C-13
21	34.7, CH_2_	3.37, d (7.1)	C-4, C-5, C-6, C-22, C-23
22	124.6, CH	5.34, overlap	C-21, C-24, C-25
23	131.4, qC	-	-
24	17.9, CH_3_	1.73, overlap	C-22, C-23, C-25
25	25.8, CH_3_	1.72, overlap	C-22, C-23, C-24
26	31.5, CH_2_	3.53, d (7.4)	C-6, C-7a, C-28
27	123.0, CH	5.43, br. t (7.4)	C-26, C-29, C-30
28	132.9, qC	-	-
29	17.9, CH_3_	1.88, s	C-27, C-28, C-30
30	25.7, CH_3_	1.82, s	C-27, C-28, C-29

^a^: Recorded at 125 MHz; ^b^: recorded at 500 MHz.

**Table 2:** Comparison of antioxidant activities of different echinulin-related alkaloids.

Compound	IC_50_ Value for DPPH Radicals (mg/mL)	Total Reducing Power (mmol/L)
8-hydroxyechinulin	0.587	0.29
echinulin	1.628	0.17
neoechinulin A	0.219	4.25

Notes: The total reducing power of different compounds was measured at a concentration of 0.5 mg/mL.

**Table 3:** Response surface test factors and horizontal design.

Factors	Level		
	−1	0	1
Methanol volume fraction (%)	60	70	80
Solid–liquid ratio (mL/g)	15	20	25
Soaking temperature (°C)	40	50	60

**Table 4:** Box–Behnken experimental design and response values.

Run	A	B	C	Extraction Yield (mg/g)
1	80	15	50	1.297
2	60	20	60	1.218
3	80	20	60	1.253
4	70	25	60	1.425
5	80	20	40	1.397
6	70	20	50	1.449
7	70	15	40	1.325
8	70	20	50	1.473
9	80	25	50	1.440
10	70	15	60	1.304
11	60	15	50	1.236
12	70	20	50	1.443
13	70	20	50	1.389
14	60	20	40	1.146
15	60	25	50	1.211
16	70	20	50	1.487
17	70	25	40	1.415

**Table 5:** ANOVA of Box–Behnken.

Source	Sum of Squares	Df	Mean Square	F-Value	*p*-Value	
Model	0.17	9	0.019	18.72	0.0004	Significant
A	0.041	1	0.041	40.28	0.0004	***
B	0.014	1	0.014	13.15	0.0084	**
C	0.0008675	1	0.0008675	0.84	0.3887	
AB	0.007048	1	0.007048	6.86	0.0344	*
AC	0.012	1	0.012	11.35	0.0119	*
BC	0.0002399	1	0.0002399	0.23	0.6437	
A^2^	0.075	1	0.075	72.62	<0.0001	***
B^2^	0.00156	1	0.00156	1.52	0.2576	
C^2^	0.016	1	0.016	15.59	0.0055	**
Residual	0.007191	7	0.001027			
Lack-of-fit	0.001568	3	0.0005227	0.37	0.7788	Not significant
Pure error	0.005624	4	0.001406			
Cor total	0.18	16				
R^2^	0.9601		R^2^_Adj_	0.9088		
C.V. %	2.38		Pred R-Squared	0.8121	Adeq Precision	12.73

Notes: Level of significance: * *p* < 0.05, ** *p* < 0.01, *** *p* < 0.001.

## Data Availability

All the data are available within the article and Supplementary Materials.

## Supplementary Materials

The following supporting information can be downloaded at: https://www.mdpi.com/article/10.3390/microorganisms12050864/s1, Figure S1: ^1^H NMR spectrum (500 MHz) of **1**; Figure S2: ^13^C NMR and DEPT spectrum (125 MHz) of **1**; Figure S3: COSY spectrum of **1**; Figure S4: HSQC spectrum of **1**; Figure S5: HMBC spectrum of **1**; Figure S6: ROESY spectrum of **1**; Figure S7: ESI–MS spectrum of **1**; Figure S8: ^1^H NMR spectrum (500 MHz) of **2**; Figure S9: ^13^C NMR and DEPT spectrum (125 MHz) of **2**; Figure S10: ESI–MS spectrum of **2**; Figure S11: ^1^H NMR spectrum (500 MHz) of **3**; Figure S12: ^13^C NMR and DEPT spectrum (125 MHz) of **3**; Figure S13: ESI–MS spectrum of **3**; Figure S14: ^1^H NMR spectrum (500 MHz) of **4**; Figure S15: ^13^C NMR and DEPT spectrum (125 MHz) of **4**; Figure S16: ESI–MS spectrum of **4**; Figure S17: ^1^H NMR spectrum (500 MHz) of **5**; Figure S18: ^13^C NMR and DEPT spectrum (125 MHz) of **5**; Figure S19: ESI–MS spectrum of **5**; Figure S20: DPPH radical scavenging capacity. (**a**) 8-hydroxyechinulin (**1**), echinulin (**3**), and neoechinulin A (**4**). (**b**) Vitamin C. Values are expressed as mean ± SD of three replicates; Table S1: NMR spectroscopic data of compound **2** in CD_3_OD (*δ* in ppm); Table S2: NMR spectroscopic data of compound **3** in CDCl_3_ (*δ* in ppm); Table S3: NMR spectroscopic data of compound **4** in CDCl_3_ (*δ* in ppm); Table S4: NMR spectroscopic data of compound **5** in DMSO-*d*_6_ (*δ* in ppm).
