# Supplementary material for: Isolation and Identification of Indole Alkaloids from Aspergillus amstelodami BSX001 and Optimization of Ultrasound-Assisted Extraction of Neoechinulin A
Source: Microorganisms. 2024 Apr 26;12(5):864. doi: 10.3390/microorganisms12050864 (PMC11123293; doi:10.3390/microorganisms12050864)
Supplement: Supplementary file 1 [file microorganisms-12-00864-s001.zip › microorganisms-2925052-supplementary.pdf]

Supplementary materials

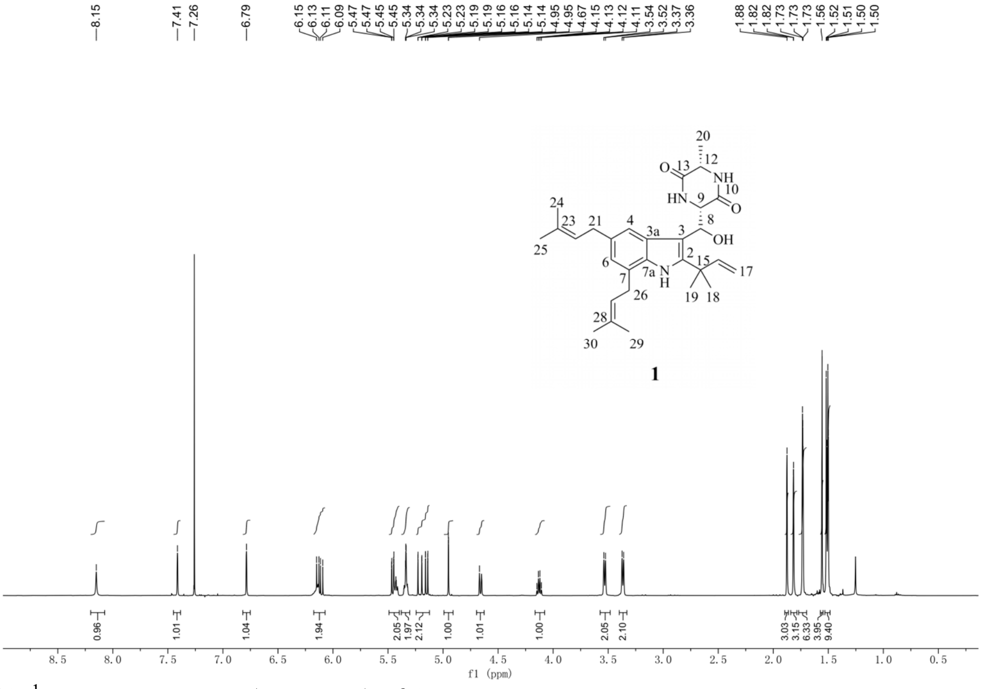

Figure S1. ^{1}H NMR spectrum (500 MHz) of **1**.

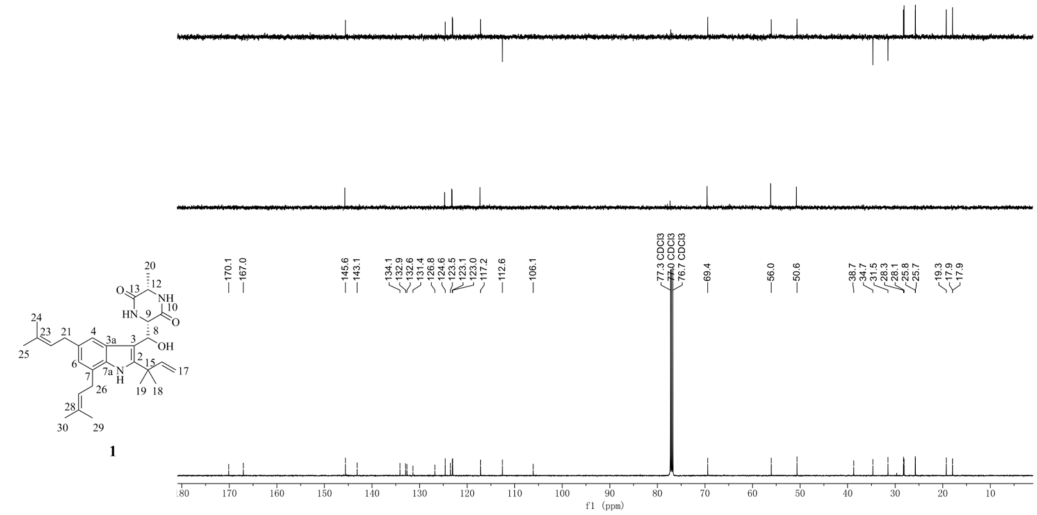

Figure S2. ^{13}C NMR and DEPT spectrum (125 MHz) of **1**.

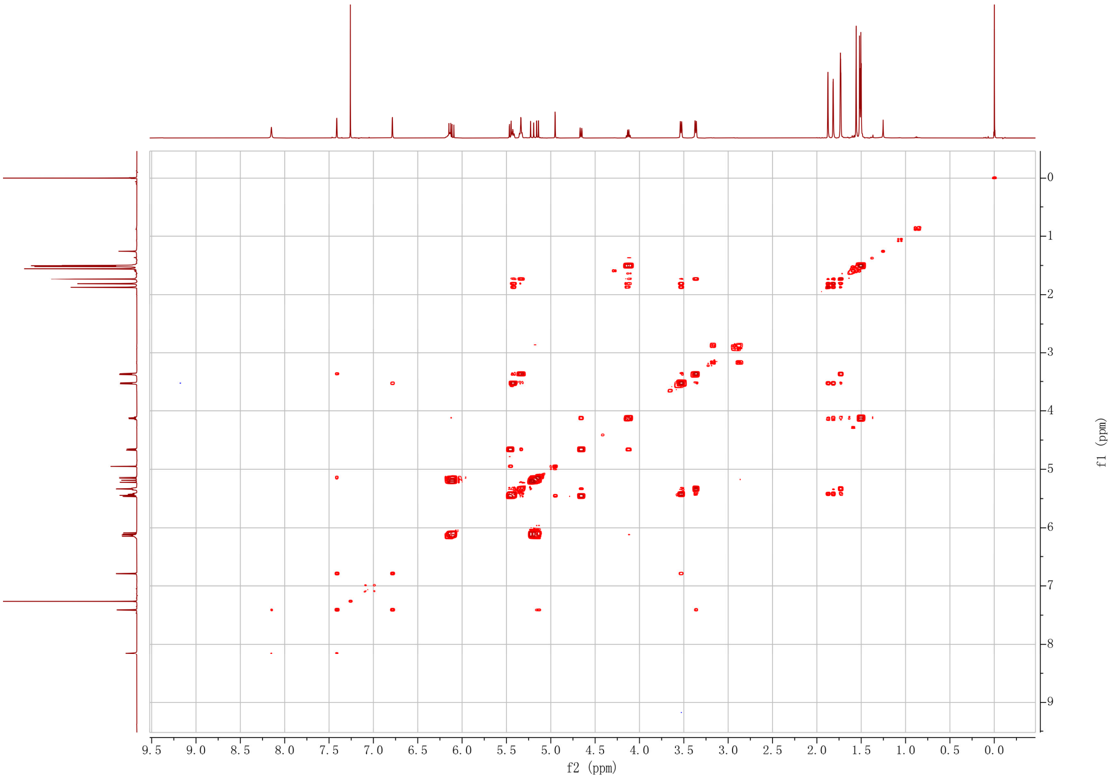

Figure S3. COSY spectrum of **1**.

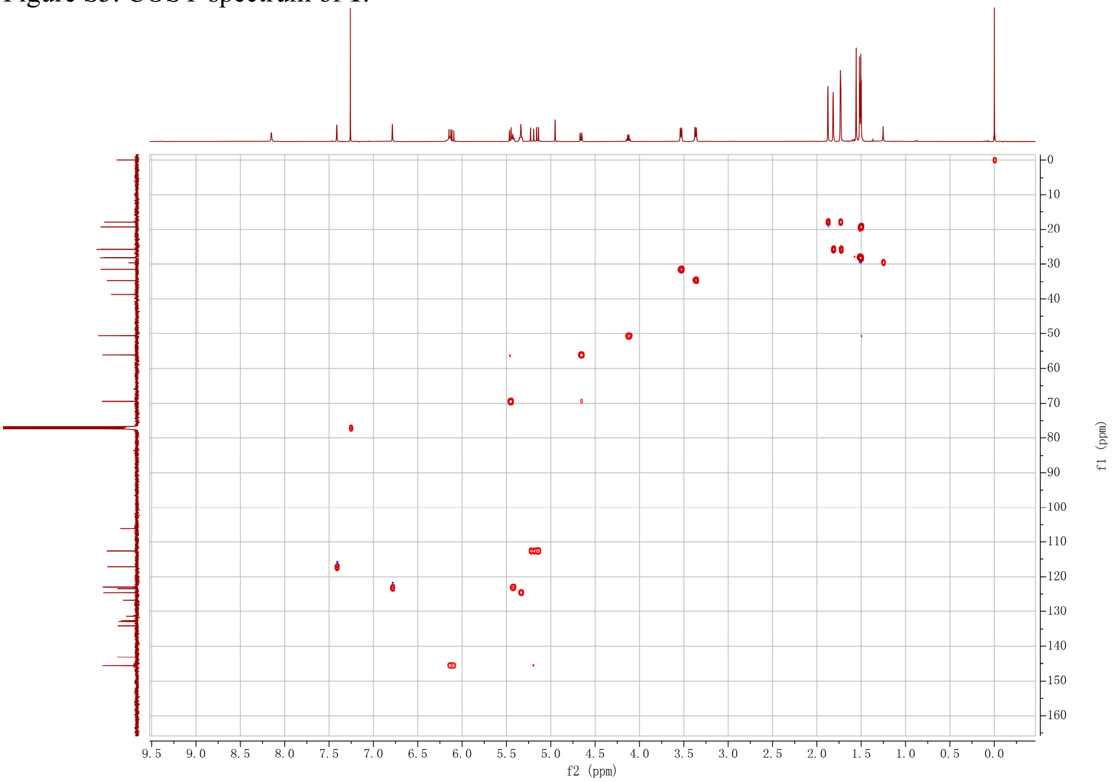

Figure S4. HSQC spectrum of **1**.

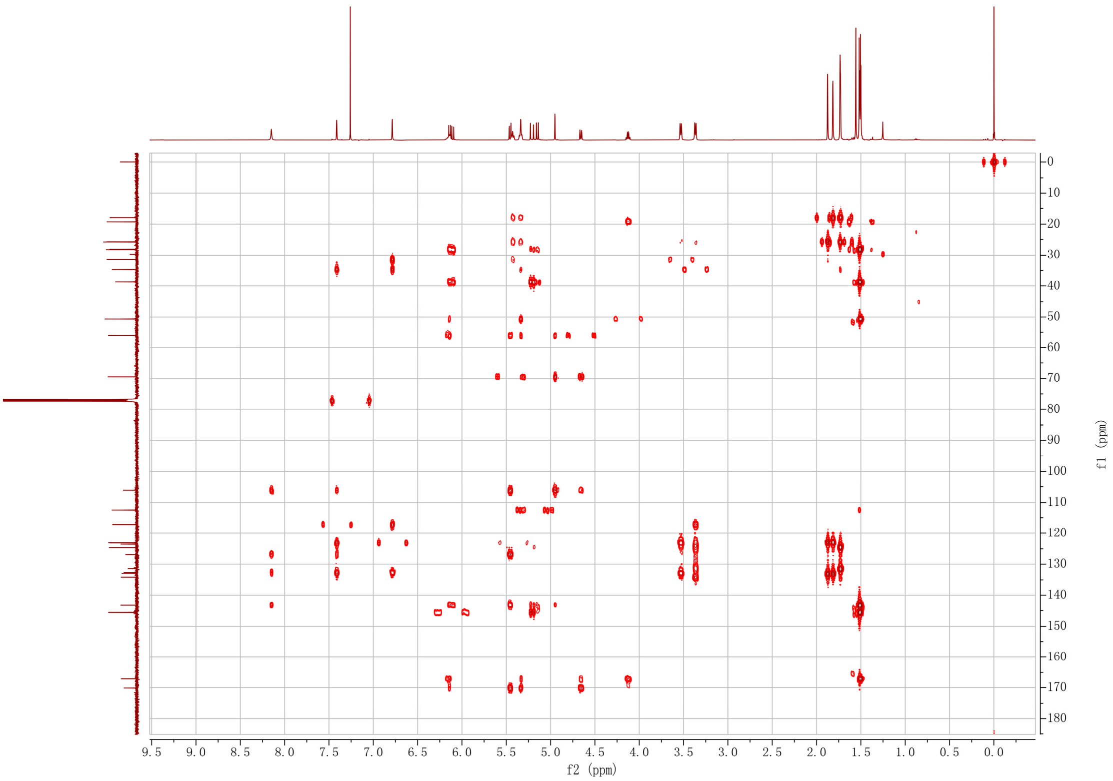

Figure S5. HMBC spectrum of **1**.

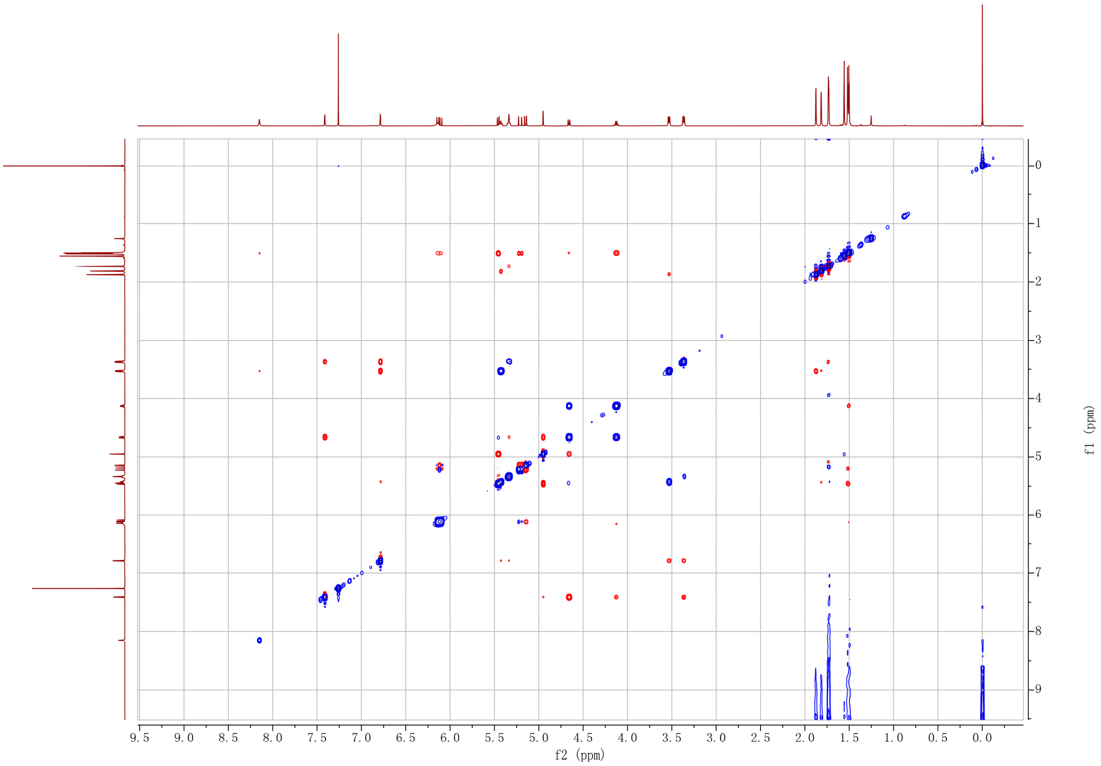

Figure S6. ROESY spectrum of **1**.

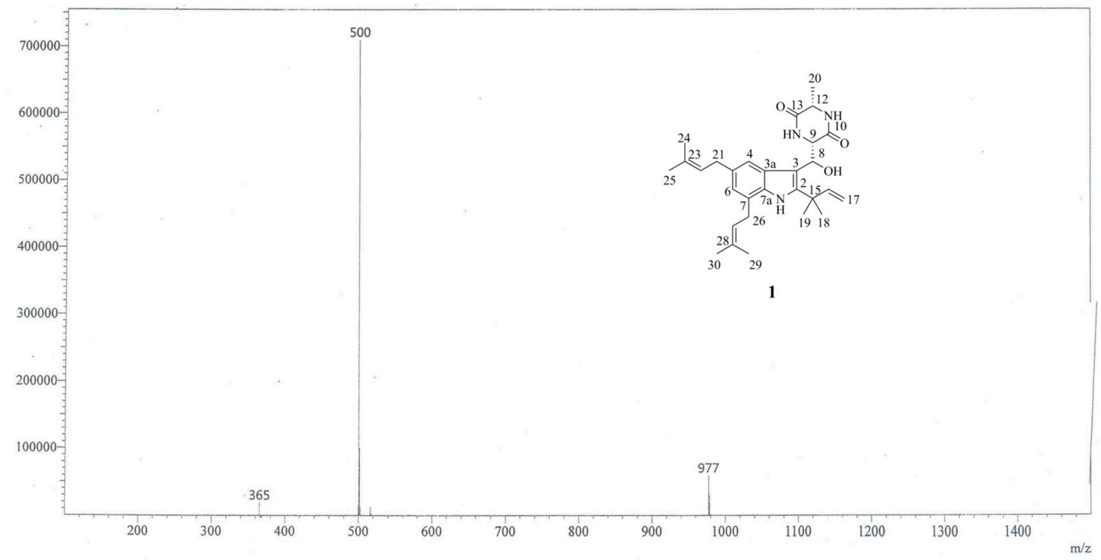

Figure S7. ESI-MS spectrum of **1**.

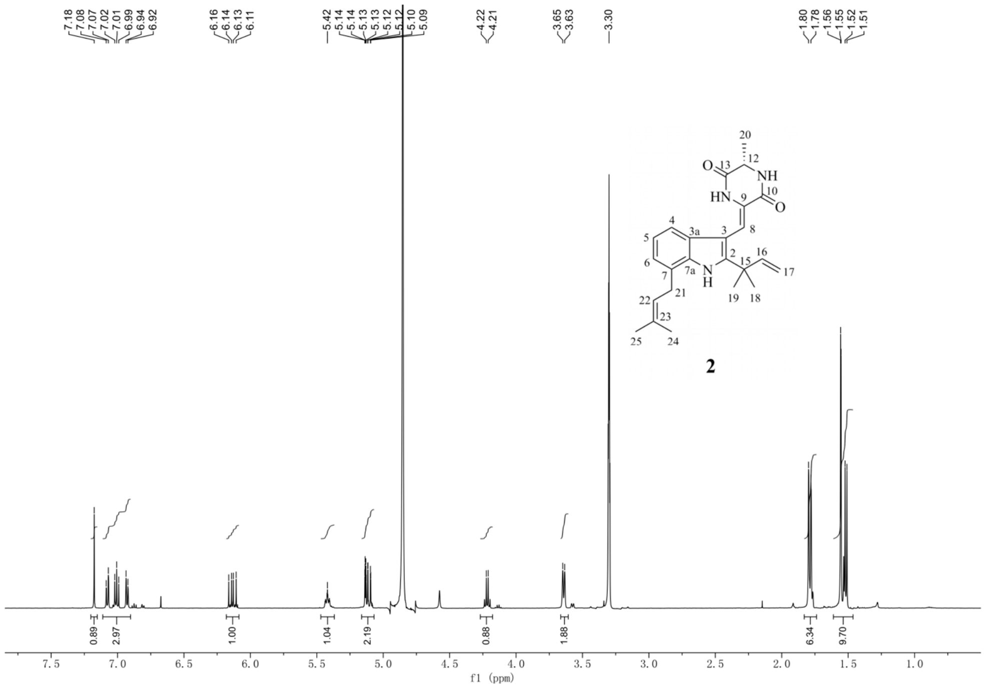

Figure S8. ^{1}H NMR spectrum (500 MHz) of **2**.

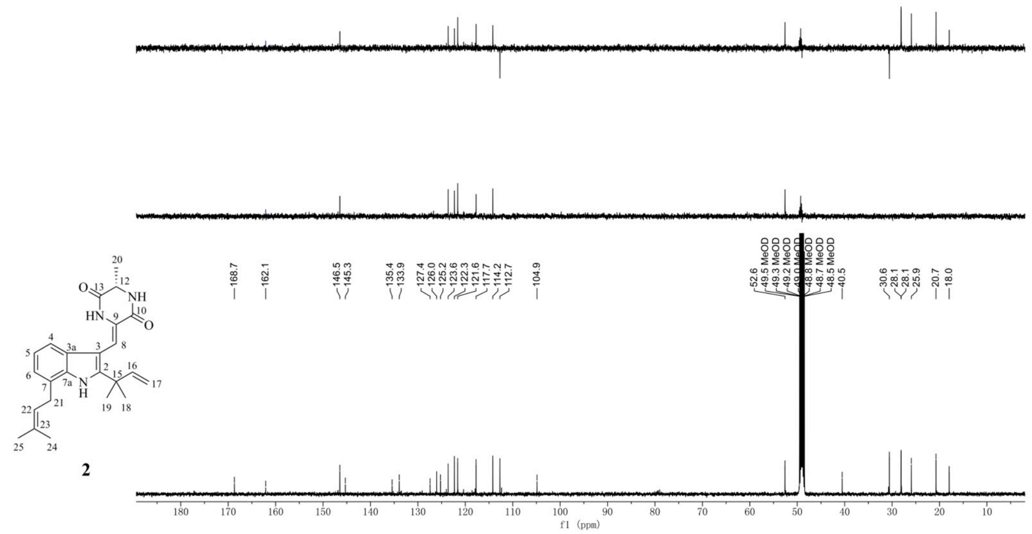

Figure S9. ^{13}C NMR and DEPT spectrum (125 MHz) of **2**.

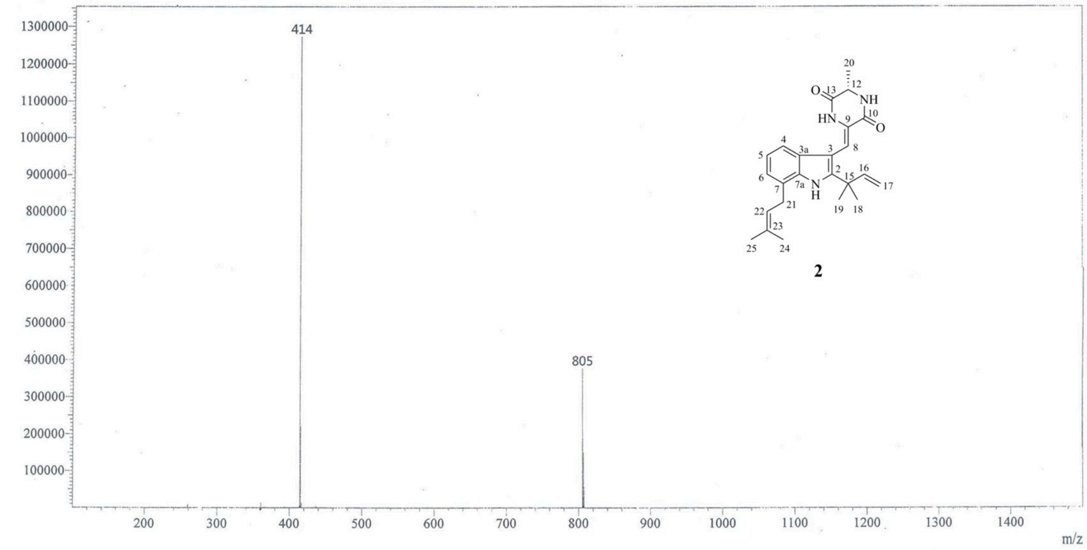

Figure S10. ESI-MS spectrum of **2**.

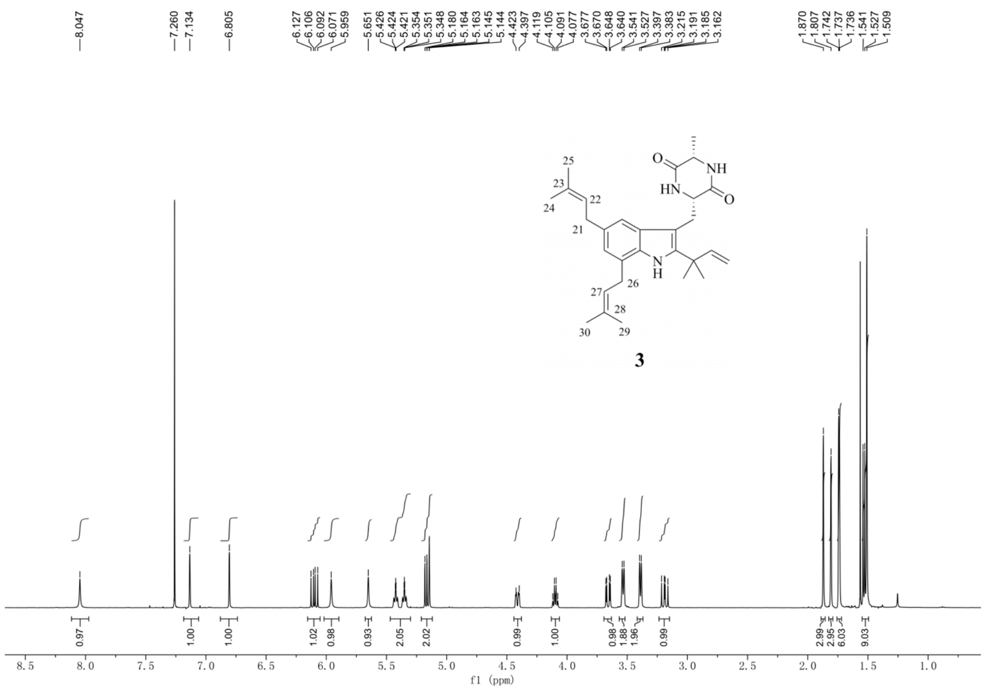

Figure S11. ^{1}H NMR spectrum (500 MHz) of **3**.

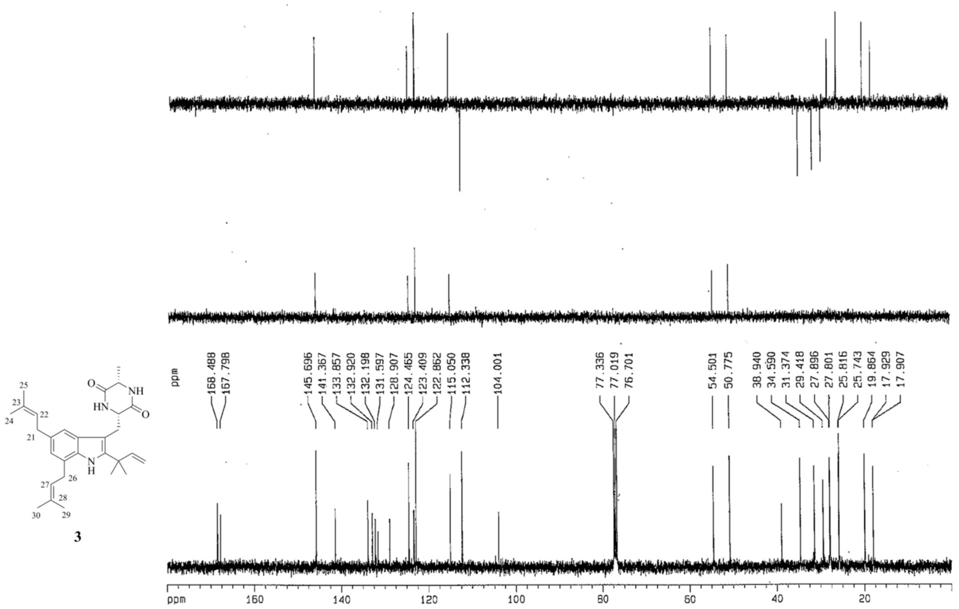

Figure S12. ^{13}C NMR and DEPT spectrum (125 MHz) of **3**.

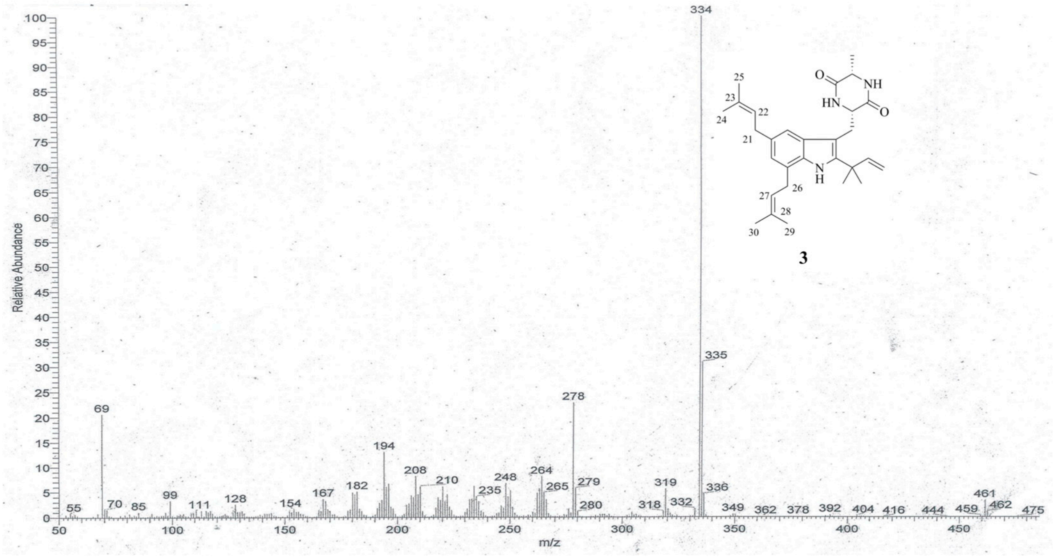

Figure S13. EI-MS spectrum of **3**.

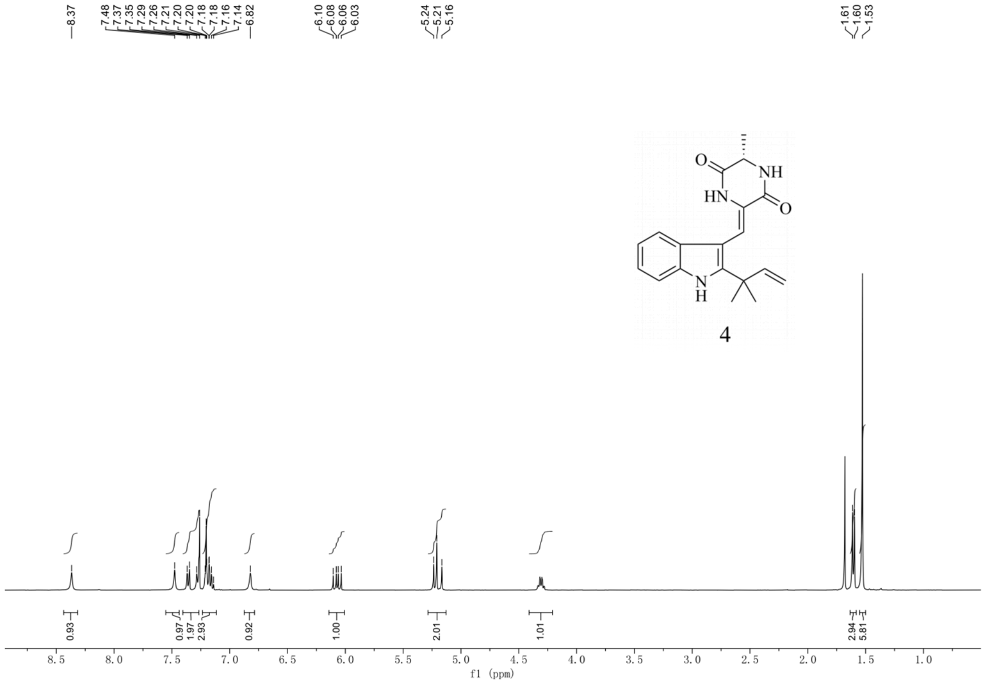

Figure S14. ^{1}H NMR spectrum (500 MHz) of **4**.

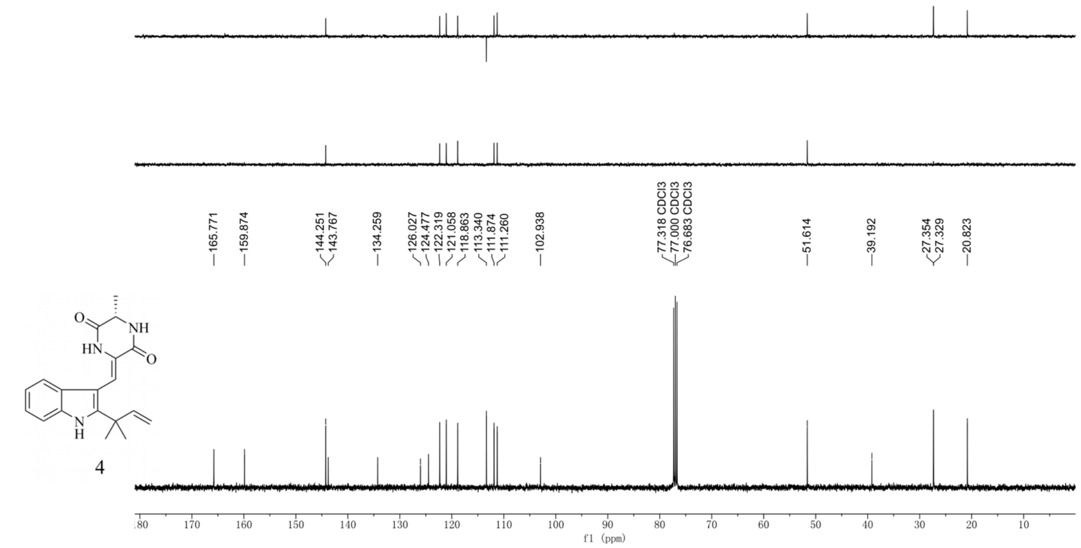

Figure S15. ^{13}C NMR and DEPT spectrum (125 MHz) of **4**.

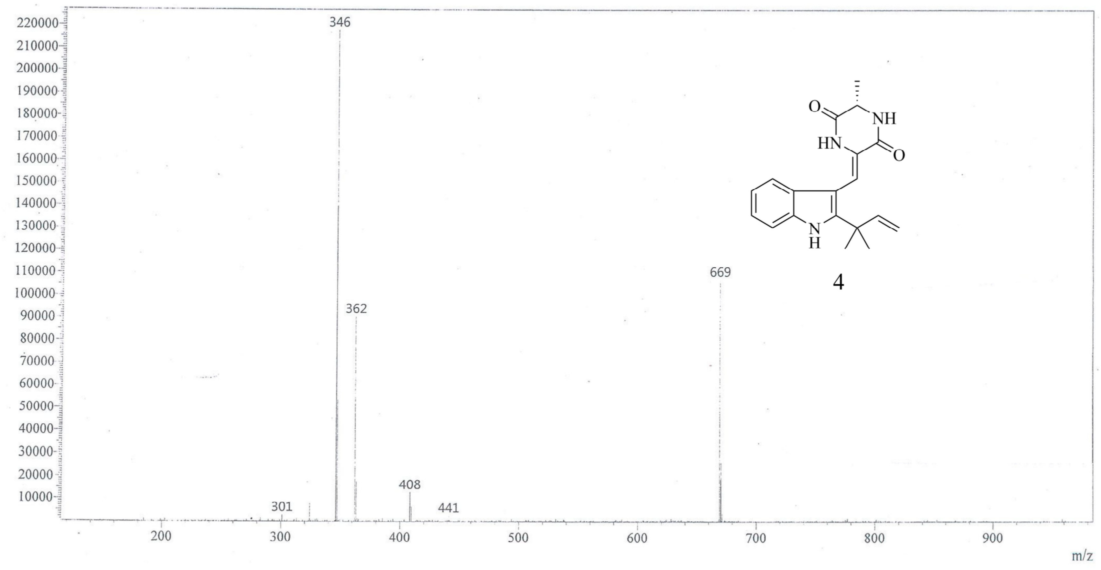

Figure S16. ESI-MS spectrum of **4**.

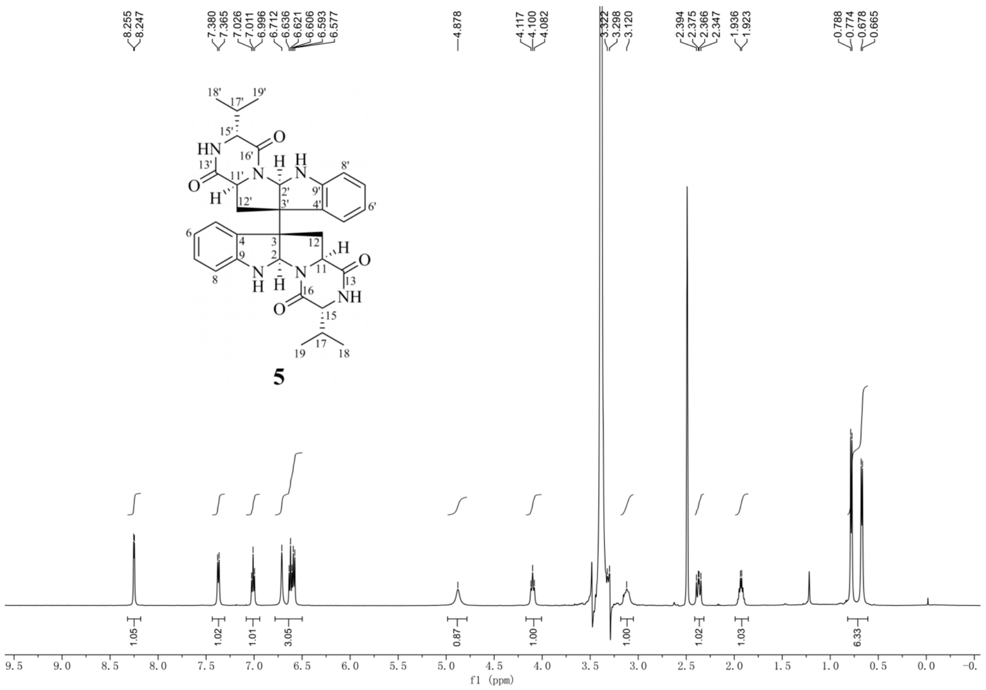

Figure S17. ^{1}H NMR spectrum (500 MHz) of **5**.

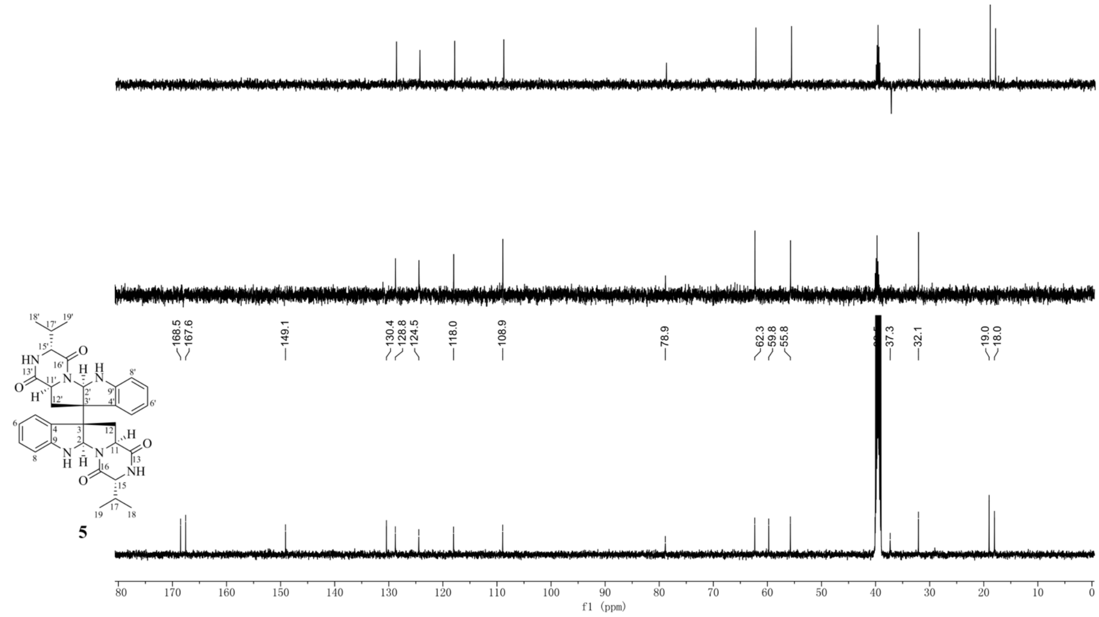

Figure S18. ^{13}C NMR and DEPT spectrum (125 MHz) of **5**.

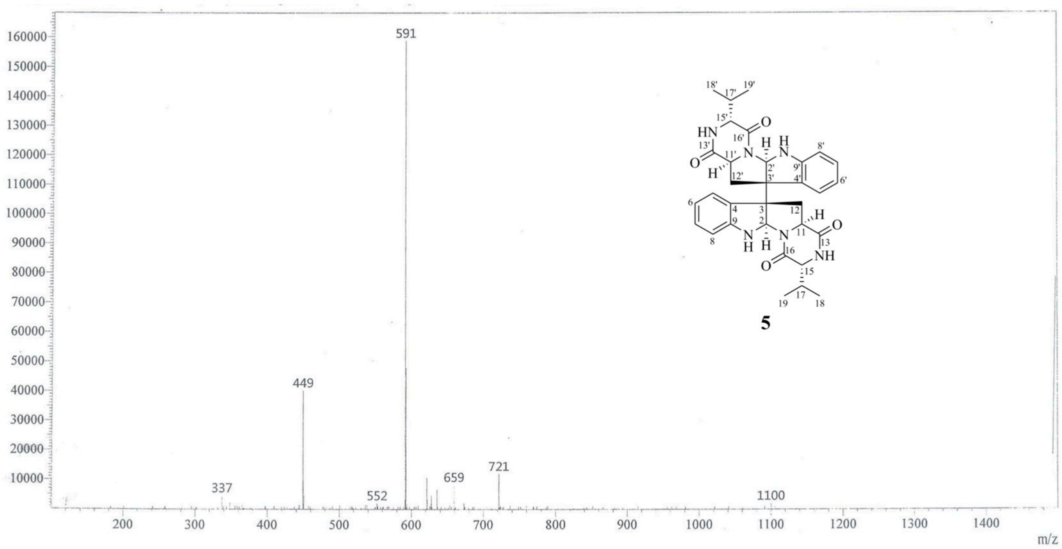

Figure S19. ESI-MS spectrum of **5**.

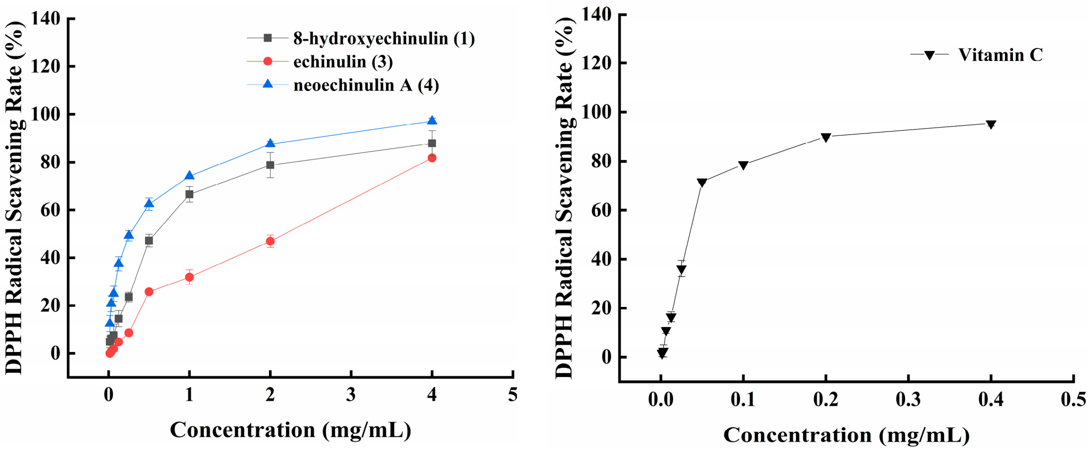

(**a**) (**b**)

Figure S20. DPPH radical scavenging capacity. (**a**) 8-hydroxyechinulin (**1**), echinulin (**3**), and neoechinulin A (**4**). (**b**) Vitamin C. Values are expressed as mean ± SD of three replicates.

Table S1. NMR spectroscopic data of compound **2** in CD_3OD (δ in ppm).

No.	δ_C[a]	δ_H[b] (J in Hz)
1-NH	-	-
2	145.3, q	-
3	104.9, q	-
3a	127.4, q	-
4	117.7, CH	7.08, br. d (7.1)
5	121.6, CH	7.01, t(7.1)
6	122.3, CH	6.93, br. d, (7.1)
7	126, q	-
7a	135.4, q	-
8	114.4, CH	7.18, s
9	125.2, q	-
10	162.1, q	-
11-NH	-	-
12	52.6, CH	4.22, q (7.0)
13	168.7, q	-
14-NH	-	-
15	40.5, q	-
16	146.5, CH	6.14, dd (17.4, 10.7)
17	112.7, CH_2	5.11, dd (17.4, 1.0)<br>5.13, dd (10.7, 1.0)
18	28.1, CH_3	1.56, s
19	28.1, CH_3	1.55, s
20	20.7, CH_3	1.52, d (7.0)
21	30.6, CH_2	3.64, d (7.3)
22	123.6, CH	5.42, m
23	133.9, q	
24	18, CH_3	1.80, br. s
25	25.9, CH_3	1.78, br. s

[a]: Recorded at 125 MHz; [b]: recorded at 500 MHz.

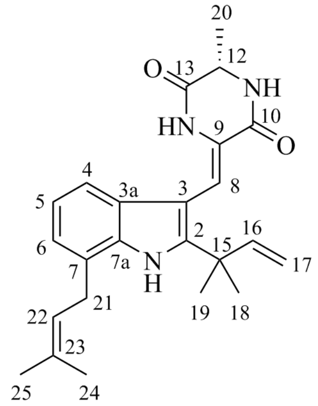

2

Table S2. NMR spectroscopic data of compound **3** in $CDCl_3$ (δ in ppm).

No.	δ_C[a]	δ_H[b] (J in Hz)
1-NH	-	-
2	141.4, qC	8.04, s
3	104, qC	-
3a	128.9, qC	-
4	115.1, CH	7.13, s
5	133.9, qC	-
6	122.9, CH	6.81, s
7	123.4, qC	-
7a	132.2, qC	-
8	29.4, CH_2	3.66, dd (14.8, 3.7)<br>3.19, dd (14.8, 11.4)
9	54.5, CH	4.41, br. dd (11.4, 3.7)
10	168.5, qC	-
11-NH		5.96, s
12	50.8, CH	4.22, q (7.0)
13	167.8, qC	-
14-NH	-	5.65, s
15	38.9, qC	-
16	145.7, CH	6.10, dd (17.4, 10.6)
17	112.3, CH_2	5.16, dd (17.4, 0.8)<br>5.15, dd (10.6, 0.8)
18	27.8, CH_3	1.51, s
19	27.9, CH_3	
20	19.9, CH_3	1.53, d (7.0)
21	34.6, CH_2	3.39, d (7.3)
22	124.5, CH	5.35, br. t (7.3)
23	131.6, qC	-
24	17.9, CH_3	1.74, br. s
25	25.7, CH_3	
26	31.4,CH_2	3.53, d (7.2)
27	122.9, CH	5.42, br. t (7.2)
28	132.9, qC	-
29	17.9, CH_3	1.87, br. s
30	25.8, CH_3	1.81, br. s

[a]: Recorded at 125 MHz; [b]: recorded at 500 MHz.

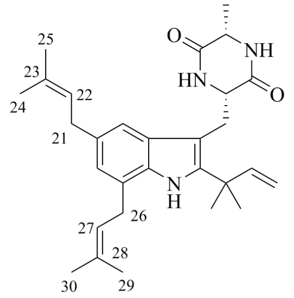

3

Table S3. NMR spectroscopic data of compound **4** in $CDCl_3$ (δ in ppm).

No.	δ_C[a]	δ_H[b] (J in Hz)
1-NH	-	8.37, s
2	143.8, qC	-
3	102.9, qC	-
3a	126, qC	-
4	118.9, CH	7.28, d (7.7)
5	121.1,CH	7.19, dd (7.7, 7.5)
6	122.3, CH	7.16, dd (7.5, 7.0)
7	111.3, qC	7.36, d (7.0)
7a	134.3, qC	-
8	111.9, CH	7.20, s
9	124.5, qC	-
10	159.8, qC	-
11-NH	-	6.82, s
12	51.7, CH	4.31, br. q (7.0)
13	165.8, qC	-
14-NH	-	7.48, s
15	39.2, qC	-
16	144.3, CH	6.07, dd (17.2, 10.5)
17	113.3,CH_2	5.22, d (10.5)<br>5.19, d (17.2)
18	27.3, CH_3	1.53, s
19	27.4, CH_3	
20	20.8, CH_3	1.61, d (7.0)

[a]: Recorded at 125 MHz; [b]: recorded at 500 MHz.

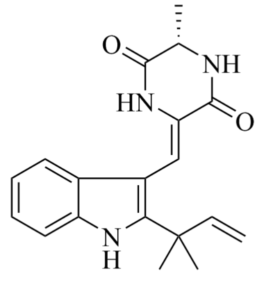

4

Table S4. NMR spectroscopic data of compound **5** in DMSO-d_6 (δ in ppm).

No.	δ_C[a]	δ_H[b] (J in Hz)
1/1'-NH	-	6.71, s
2/2'	78.9, CH	4.88, br. s
3/3'	59.7, qC	-
4/4'	130.4, qC	-
5/5'	124.5, CH	7.37, d (7.5)
6/6'	118.0, CH	6.62, t (7.5)
7/7'	128.8, CH	7.01, t (7.5)
8/8'	108.9, CH	6.59, d (7.5)
9/9'	149.1, qC	-
10/10'-N	-	-
11/11'	55.8, CH	4.10, t (8.7)
12/12'	37.3, CH_2	3.12, m
13/13'	168.5, qC	-
14/14'-NH	-	8.25, d (4.2)
15/15'	62.3, CH	3.37, overlap<br>2.37, d (14.0, 9.4)
16/16'	167.6, qC	-
17/17'	32.0, CH	1.93, m
18/18'	19.0, CH_3	0.78, d (6.8)
19/19'	18.0, CH_3	0.67, d (6.6)

[a]: Recorded at 125 MHz; [b]: recorded at 500 MHz.

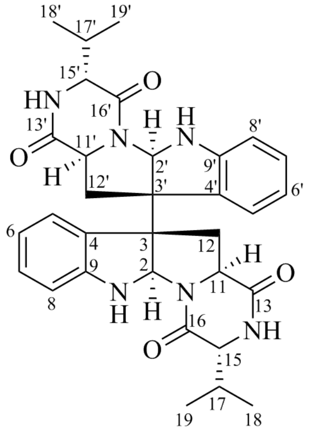

5
